# Hospital and Institutional News

**Published:** 1919-07-05

**Authors:** 


### HOSPITAL AND INSTITUTIONAL NEWS.

### THE MINISTRY OF HEALTH.

WHEN the Local Government Board ceased to exist at the end of last month, all the duties pertaining to the office of President of that Board passed automatically to the Minister of Health, who is now empowered to transfer to other Departments such duties and powers as are not incidental to the health of the people. We may see many changes and considerable reorganisation, but those who expect developments of unusual swiftness must remember that these transfers will be made from time to time by Orders in Council—and that such cannot become immediately operative; they must lie on the table of both Houses of Parliament for not less than thirty days, and must be approved of by both Houses. In identical fashion several matters affecting public health but now vested in other existing Departments will be transferred to the Ministry of Health, and among them will fall care of nursing and expectant mothers, infant welfare, lunacy, mental deficiency, and regulation of midwifery. Recently Dr. Addison stated that, with a view to all-round improvement, many proposals will be submitted immediately to the four consultative councils already organised, and it is hoped to get many of these proposals well on the way this year. Much can be done without waiting for further legislative authority, and everything possible will be proceeded with after the minimum of delay. We are in receipt of two circulars, one referring to communications regarding business of approved societies in England, which should now be addressed to the Controller, Health Insurance Department, Ministry of Health, at Wellington House, or at the branch offices which have previously dealt with the particular matters in question. The second states that all communications upon the business of Insurance Committees in England must now be directed to the Secretary, Ministry of Health, and addressed, for the time being, to Wellington House, Buckingham Gate, S.W. 1.

### WAR OFFICE AND DOCTORS.

THE War Office announces that it is prepared again to accept the services of medical practitioners under the following terms: Candidates who have served before will be commissioned in their previous rank, otherwise they will rank as lieutenants. Period of engagement is to be for one year, or until no longer required, which ever shall first happen. Pay will be at the rate of £550 a year, inclusive of all allowances and rations, or the allowance in lieu thereof. Outfit and kit allowance will be issued to candidates who have not received them for previous service. All demobilised officers are eligible to re-engage under these terms, but must be fit for general service at home or abroad. Applications should be addressed to the Secretary, War Office, Adastral House, London, E.C. 4.

### THE CARE OF FRACTURES IN CIVIL HOSPITALS.

THE war has been responsible for greater advances in the treatment of compound fractures than had been made since Listerian principles revolutionised previous surgical practice. Compound fractures produced by gunshot wound differ from the ordinary cases of civil life in certain respects; but the lessons learned during the war apply, with hardly any modification, to the accident cases of peace. On p. 347 will be found an important discussion by Major Hey Groves of the treatment of gunshot fractures of the thigh bone, concluding with some very pregnant suggestions for turning war experience to good effect in civil hospitals. It is too true, as he affirms, that in many general hospitals little interest is taken in fracture cases, and that the results are not so good as they might be and ought to be. This is partly the fault of the teaching staffs, who are seldom called in to cases of fracture in private practice, and so devote more attention in hospital to abdominal operations, which bring more *prestige*, and therefore more consulting practice: another drawback of the system by which medical teachers are not paid a living wage for teaching, but must earn their bread by converting their clinics into advertisements for consulting practice. We commend Mr. Groves's remarks on this subject to the earnest attention of institutional workers.

### SIR D. HAIG AND MEDICAL BOARDS.

SIR DOUGLAS HAIG'S evidence before the Select Committee on Pensions, given at the House of Commons on July 1, was evidently inspired by strong, even fervid, conviction. That he is acquainted with numerous pension grievances, as to which his help and advice have been solicited, is indubitable; nor does anyone who knows the facts regard the Pensions Ministry as impeccable. But at the same time some of Sir Douglas's suggestions betray imperfect acquaintance with important scientific and medical technicalities; this is no blame to him, for he has had no time to give attention to them. Thus his strongly worded criticisms of Medical Boards can doubtless be justified by individual cases of error: that some of these Boards include ignorant, some unsympathetic, and some ungenerous doctors is true enough, but to brand them all with those defects is not only unjust, it is untrue. Nor does the suggestion that a combatant (non-medical) officer should sit on each board as an assessor seem to us a practicable idea. The boards are there not only to serve the disabled soldier, but also to protect the taxpayer from fraud, which is not at all rarely attempted. Again, Sir Douglas's remarks about sufferers from neurasthenia, shell-shock, and gas-poisoning betray lack of knowledge of these cases; in the great bulk of them to betray lavish sympathy is to delay the cure of the patient and his return to useful productive life; and, again, it is particularly in this connection that malingering is rife and difficult to detect.

### PRESENTATION TO SIR WM. OSLER.

ON Friday, July 11, at 5.15 P.M., the eve of his seventieth birthday, an Anniversary Book written and inscribed by many of his pupils, colleagues, and friends is to be presented to Sir William Osler, Regius Professor of Medicine at the University of Oxford. Sir Clifford Allbutt will make the presentation, which is to take place in the library of the Royal Society of Medicine, London, and all members of the medical profession are invited to be present. The book will consist of two large octavo volumes, reproducing as nearly as possible the printer's and binder's arts of the fifteenth century. The frontispiece is a very fine steel engraving of Sir Wm. Osler, and there will be about one hundred contributors, English and American, represented in the text. There will be only one printing of the book, which will not be publicly advertised, and the supply of copies is limited to those who forward the subscription of two guineas in advance to Messrs. H. K. Lewis and Co.

### ARMY NURSES AND THE R.A.M.C. WAR MEMORIAL.

A MOVEMENT has been started amongst the members of the Royal Army Medical Corps to create a memorial to those of their comrades who have fallen in the late wars. The proposal has been received with enthusiasm, contributions have been flowing in, and all ranks have shown a wish to take part in the construction arrangements. This is proper and in accordance with good tradition. A question has arisen, however, as to whether the memorial shall be confined strictly to the Royal Army Medical Corps, or whether it shall include the associated services: the nurses, and the members of the voluntary-aid detachments, the St. John's Ambulance Association, and the Red Cross Society. The affair is one of sentiment, and whatever decision is made will depend upon and will represent the sentiment of the day. This being so, if opinion be allowed free play among all who are concerned and can express their views, we believe that a desire will be remarked to provide a wide and generous scope for the memorial; and that it will be arranged to comprise, both in its objects and the means by which these are to be obtained, all who have worked together in caring for the sick and wounded during the past years of strain and battle. The nurses have shown a courage in danger and an endurance through hardship which surpass the imagination and cannot be conveyed in words. Their spirits have conquered weariness, their enthusiasm throughout has never faltered. In the darkest hours they have cheered and comforted the sick and broken men. Their devotion has been one of the contributory causes of our ultimate victory and the emancipation of the world. Not a soldier faced the enemy but could feel that not far behind the lines were loving hearts and gentle hands to tend him if he fell. Who shall measure the influence of such thoughts; who shall doubt or deny the great part they took in upholding to the end the unsurpassed moral of our fighting forces? Many of these good women have lost their lives—by the sinking of ships at sea, by bombs and shells falling into hospitals, by disease. Their inclusion in any memorial founded by the Royal Army Medical Corps would be graceful and honourable. For these reasons we hope that those who are responsible for the memorial will make arrangements to widen its field in the manner that has been suggested.

### PEACE CELEBRATIONS: A SUGGESTION.

THE popularity of the Oxford Pageant of Victory is gratifying, not only because Oxford is a fine centre for such a display, but because it is designed to benefit the Radcliffe Infirmary. The League of Mercy is another beneficiary. Perhaps other historic cities may like to group their peace celebrations round a similar pageant for the benefit of their local hospitals. If not, it is to be feared that such celebrations will often be formless.

### GROUPED CONVALESCENT HOMES.

THE war has accustomed us to the plan of grouping minor institutions round a mother hospital, and we hope that it is not too late for the idea to bear fruit in civilian hospitals. With the severe shortage of beds, and the long lists of waiting cases, what is wanted immediately is the provision of several convalescent homes for each big voluntary hospital. Where this idea is put in practice it should be possible to reduce the average length of stay of an in-patient to fourteen days. There are still many houses which have proved their value during the war for hospital work, viz., the discharged V.A.D.s, and there must be many who would wish to continue though preferably under less severe conditions than were the rule for them in military hospitals. Under a capable matron they should be able to do the necessary work for convalescents, and would find it lighter than the work expected of them in the war. Since it was the custom to support hospital work in these same houses during the war, we believe that the money could largely be raised locally, and that by this plan much could be done to supply the extra beds before building can be completed or even undertaken. The Royal Victoria Infirmary, Newcastle, is considering this plan, which might well be made general.

### THE MEDICAL MISSIONARY EXHIBITION.

IT is always good to hear of anything which lifts the veil from the work of medical missions, and we hope that the United Medical Missions Exhibition, which is open for a (rapidly diminishing) fortnight in the crypt grounds of St. Martin's-in-the-Fields, will awaken public interest. The crypt (it is amusing to notice that the spot chosen for the exhibition is a crypt) is full of curios from distant parts of the world, and the inspection of these is enlivened by periodic lectures. Medical missions are so important that we often wonder why those responsible for them do not seem more interested in the curious experiences and invaluable results of the work.

### A FLOATING MEDICAL SCHOOL.

DR. LOUIS SAMBON, who has for so long been identified with the study of tropical medicine, has made a suggestion which should interest workers in tropical medicine in this country and elsewhere. He proposes the establishment of a floating school for the study of these diseases. The idea is that the students shall embark in a ship fitted with all the requisite apparatus and laboratories, and shall be taken from one tropical area to another. Thus they will be enabled to visit the many fields in which disease flourishes, and, as the passengers will consist of men of every nationality, a new link of world friendship will be forged. Moreover, those workers who are struggling against the various plagues of hot climates will be cheered and stimulated by the visits of the floating school. It is intended to visit British, French, American, and Italian possessions—indeed, to make the scholars in a true sense citizens of the world. So far the scheme has been enthusiastically received, and if the difficulty of the great shortage of ships can be surmounted, should be carried to a successful conclusion. Dr. Andrew Balfour suggested the same idea some years ago in connection with Egypt, but nothing came of it at the time.

### A WELSH BOARD OF HEALTH.

It is announced that a Board of Health for Wales is to be quickly established, to consist of the three Welsh Insurance Commissioners, Sir Thomas Hughes, Dr. Meredith Richards, and Mr. J. Rowland, C.B.E. The Cardiff Medical School and the Welsh National Memorial are also expected to be represented upon the new board.

### CONTROL OF PICTURE PALACES.

With a view to minimising the danger of the spread of infection and injury to the eyes of children, the Leicester Sanitary Committee, acting upon a suggestion made to Dr. C. Killick-Millard, M.O.H., by Dr. Warner, school medical officer, have recently approached the Watch Committee, asking the latter to consider the practicability of duplicating children's performances at moving picture palaces and at the same time reducing the duration of the performance and the number of children allowed to be present at a time by one-half. Dr. Millard writes: "As one hour's entertainment for one penny is extremely good value, it is unlikely that the total attendance would be materially affected, so that the proprietors should not suffer financially and but little extra trouble would be involved. A short interval between the performances should be insisted upon, during which the buildings should be thoroughly aired." The point is well worth consideration, for there is no doubt that the crowded cinema has already proved to be a most potent factor in dissemination of disease.

### A NEW HOSPITAL FOR GLASGOW.

The scheme to rebuild the Glasgow Hospital for Diseases of the Ear, Throat and Nose on a new spot, which was approved a year ago, has now led to the purchase of a fresh site. The new property consists of 303-308 St. Vincent Street, where there is space for an institution of at least forty beds, an out-patient department, and also a nurses' home.

### THE QUEEN'S APPRECIATION.

A short time ago the King, the Queen and Princess Mary visited the Charterhouse Military Hospital and watched with interest a number of armless men at work. Her Majesty was struck with the work of Private J. Preston, and expressed a wish to possess the inlaid tray which he was making. In due course the tray, beautifully polished, and bearing a suitable inscription, was sent to the Queen, whose private secretary has written to Mr. Fred Bonsor, the Chairman, as follows: "I am commanded by the Queen to acknowledge the tray which is the work of Private J. Preston. Her Majesty remembers very well seeing him at work, and I am to ask if you will be so good as to express to him her Majesty's sincere thanks for this interesting specimen of his work, which it gives the Queen great pleasure to accept."

### THE GIFT OF A SITE AT PETERBOROUGH.

The proposal for a War Memorial Hospital at Peterborough, which some think too ambitious a scheme, and others say has hung fire too long, has now received a powerful backing from Mr. John Bunting, of Spalding. He has given £5,000 to purchase a site, and the sites committee is now considering three sites which are eligible. With such a help onwards, we hope that the inhabitants of Peterborough will quickly do their share in guaranteeing its maintenance, and that the richer folk will contribute towards the cost of the building. It rests with the people themselves to disprove the pessimistic fears of Mr. J. H. Beeby, one of the three trustees of the existing hospital.

### A HELD-UP NURSES' HOME.

The generous gift of £10,000 to build a nurses' home at the Boscombe branch of the Royal Victoria and West Hants Hospital cannot be translated into bricks and mortar at present. Mr. Walter Wild Clark, the donor, has found, like other intending builders, that it is difficult, if not impossible, to get tenders. The increase in the cost of labour and of materials, and the uncertainty as to when these increases will stop, prevent builders from tendering at all. They will not bind themselves by an estimate. It is probably safe to expect the cost to be twice as much as is anticipated, so that at the very moment, when the demand for building is at its height, no work can be decided on; and this not because of restrictions or absence of material or labour so much as through uncertainty and dismay. It is very hard upon hospital committees and architects who have plans drawn and funds in hand.

### COST OF TREATMENT FOR SCHOOLCHILDREN.

The London County Council Education Committee have made provision for the medical and dental treatment of 185,438 schoolchildren during the year 1919-20. The cost of the arrangements (apart from administrative charges) is stated to be £52,459, as compared with £48,823 authorised for 1918-19. Owing to the additional expenditure the Finance Committee reporting on this subject say the question arises whether the charges at present made should not now be increased, having regard, on the one hand, to the general increase in wages and the larger ability of parents to pay, and, on the other, to the increased cost of the medical service. According to the estimates for the current financial year, the cost of medical treatment of children in elementary schools is £74,260, while the amount estimated to be recovered is only £2,700.

### A FORTY-EIGHT-HOUR WEEK IN INSTITUTIONS.

An interesting report on the hours of duty of institutional officers has been drafted by the Advisory Committee of the Birmingham Board of Guardians. Investigation shows that the present hours of duty greatly vary in the different classes of officers, the extremes being from forty-seven in the case of non-residents to eighty for some resident attendants. In endeavouring to arrange a forty-eight-hour week, the committee recommends that officers employed under trade-union conditions shall work for hours corresponding to those fixed for their particular trade; that so far as possible the hours of duty of general assistant institutional officers and the normal staff at the Union offices shall be forty-eight per week, and that where this is impracticable, because of the nature of the duties or other special reason, appropriate arrangements shall be made; that meal-times shall not be included in the hours of duty; and that a sufficient staff shall be engaged to prevent overtime, save on emergency, since the object of the forty-eight-hour week is not to increase the earnings but to secure the leisure of the staff. The earliest date for introducing the forty-eight-hour week is to be reported to the Advisory Committee by the committees of the different institutions. How far such institutions will lead to the introduction of a statutory week in hospitals remains to be seen; but the tendency is general, and not nurses alone probably will be touched by it.

### PROPOSED NEW CHESHIRE SANATORIUM.

Warrington Corporation has purchased Hefferston Grange, Weaverham, ten miles from the town, together with an estate of about 100 acres. This fine old Georgian house, part of which dates back to 1741, stands on the site of a much older building that belonged to the monks of Vale Royal Abbey. It is accessible, though well away from the main high roads, and situated in the centre of Cheshire, with extensive views of the surrounding country. The intention is to use this site for a sanatorium. The Grange is well adapted for an administrative block, whilst hospital buildings for at least seventy patients will be erected in the grounds. It is hoped that the necessary work will be carried out this summer, so that the institution can be opened later in the year.

### THE NEW LANCASHIRE SANATORIUM.

We are glad to see that the Preston Corporation has set an example by purchasing the estate known as The Chestnuts, Ribbleton, for patients in an advanced stage of consumption. Under Mr. Henry Cartmell, the board of management will consist of six members of the Health Committee, and six members of the Preston Insurance Committee. The matron is Miss Wyrill, and provision is made for fourteen patients. When the sanatorium was opened Dr. R. C. Brown referred to the high rate of mortality from consumption in Lancashire and Preston. He then discussed the open-air treatment, which he said was also the great remedy for cough. For prevention, the detection of early cases is the need. But at present, and along with it, there is an equal need for sanatoria for advanced cases, who at present are perhaps the most unfortunate of patients on the waiting-list of any institutions.

### COTTAGE HOSPITAL POUND DAYS.

No method of raising money is so generally understood as to be free from misapprehension, and we learn that Hornsey Cottage Hospital has benefited by changing its pound day into "pence day," because people are no longer frightened at the sum of money or weight in kind which they are expected to bring. A cottage hospital still suggests a rural institution, with supporters in the neighbouring country folk. But "cottage hospital" has ceased to be a term of precision, and pound day in a suburb or a city must expect rather to be honoured in pence. For this reason other cottage hospitals in populous districts may like to know a simple means of improving the annual yield from this source.

### BUSHEY PARK CHILDREN'S SANATORIUM.

The King and Queen have generously offered to lend the well-known house, Upper Lodge, in Bushey Park, for ten years as a camp sanatorium for delicate London children, under the London County Council. The huts and buildings in Bushey Park have been presented to the Council by the Canadian Red Cross Society. The buildings will include a dental clinic, and special teachers will be engaged to continue the children's education during their month's visit to the sanatorium school. The number to be accommodated will be 300, and it is hoped to secure other camps in North, South and East London.

### THIS WEEK'S DRUG MARKET.

Business is good, and signs are not wanting that it will continue to get better. The signing of Peace has cleared the air, but it still remains for the Government to declare its trade policy. So far as the drug market is concerned, the doubtful factor is Germany's stocks of drugs and her potential output. Until we have some definite notion as to how, when, and where these stocks are to be unloaded, we must still be unable to form any just estimate as to the future trend of prices. In the meantime, the downward movement is far from being general, and is much slower. Camphor continues to move upwards in price. The values of menthol and peppermint oil are firmly maintained. Saffron is dearer. The position of opium is practically unchanged, but we are almost sure to see some decided movement in this market before long. Star aniseed oil has an upward price tendency. The price of cocaine is not stable, and with a slow demand the price is not firmly maintained. In synthetic drugs business is on the quiet side; and, on the whole, prices are inclined downwards.

